# Corneal Cross-Linking: The Evolution of Treatment for Corneal Diseases

**DOI:** 10.3389/fphar.2021.686630

**Published:** 2021-07-19

**Authors:** Duoduo Wu, Dawn Ka-Ann Lim, Blanche Xiao Hong Lim, Nathan Wong, Farhad Hafezi, Ray Manotosh, Chris Hong Long Lim

**Affiliations:** ^1^ Yong Loo Lin School of Medicine, National University of Singapore, Singapore, Singapore; ^2^ Department of Ophthalmology, National University Health System, Singapore, Singapore; ^3^ Royal Victorian Eye Hospital, Melbourne, VIC, Australia; ^4^ Ocular Cell Biology Group, Center for Applied Biotechnology and Molecular Medicine, University of Zurich, Zurich, Switzerland; ^5^ ELZA Institute, Dietikon, Switzerland; ^6^ Faculty of Medicine, University of Geneva, Geneva, Switzerland; ^7^ Ophthalmology, USC Roski Eye Institute, Los Angeles, CA, United States; ^8^ Ophthalmology, Wenzhou Medical University, Wenzhou, China; ^9^ Singapore Eye Research Institute, Singapore, Singapore; ^10^ School of Optometry and Vision Science, University of New South Wales, Sydney, NSW, Australia

**Keywords:** corneal cross-linking, customised CXL, infectious keratitis, keratoconus, myopia

## Abstract

Corneal cross-linking (CXL) using riboflavin and ultraviolet A (UVA) light has become a useful treatment option for not only corneal ectasias, such as keratoconus, but also a number of other corneal diseases. Riboflavin is a photoactivated chromophore that plays an integral role in facilitating collagen crosslinking. Modifications to its formulation and administration have been proposed to overcome shortcomings of the original epithelium-off Dresden CXL protocol and increase its applicability across various clinical scenarios. Hypoosmolar riboflavin formulations have been used to artificially thicken thin corneas prior to cross-linking to mitigate safety concerns regarding the corneal endothelium, whereas hyperosmolar formulations have been used to reduce corneal oedema when treating bullous keratopathy. Transepithelial protocols incorporate supplementary topical medications such as tetracaine, benzalkonium chloride, ethylenediaminetetraacetic acid and trometamol to disrupt the corneal epithelium and improve corneal penetration of riboflavin. Further assistive techniques include use of iontophoresis and other wearable adjuncts to facilitate epithelium-on riboflavin administration. Recent advances include, Photoactivated Chromophore for Keratitis-Corneal Cross-linking (PACK-CXL) for treatment of infectious keratitis, customised protocols (CurV) utilising riboflavin coupled with customised UVA shapes to induce targeted stiffening have further induced interest in the field. This review aims to examine the latest advances in riboflavin and UVA administration, and their efficacy and safety in treating a range of corneal diseases. With such diverse riboflavin delivery options, CXL is well primed to complement the armamentarium of therapeutic options available for the treatment of a variety of corneal diseases.

## Introduction

Corneal ectasias like keratoconus, pellucid marginal degeneration, and post-refractory or post-traumatic corneal ectasia are characterised by progressive corneal steepening, thinning and refractive changes. These disorders can severely impede an individual’s sight and quality of life and are potentially disabling.

Conventional non-surgical management of corneal ectasias consists of conservative options such as spectacles and contact lenses. Hard contact lenses, such as scleral or hybrid contact lenses, may be prescribed. Classic surgical options include penetrating keratoplasty, deep anterior lamellar keratoplasty or intracorneal ring segments. More recently, techniques such as the use of isolated Bowman layer transplantation and corneal allogenic intrastromal ring segment (CAIRS) implantation have been reported as effective techniques in the management of patients with keratoconus (Jacob et al., 2018; van Dijk et al., 2018; Zygoura et al., 2018). Although such approaches can be effective, they can be costly, and dependent upon skilled surgeons for good outcomes. Furthermore, corneal grafting requires patients to take long-term immunosuppressant drugs, and the procedure may place patients at an increased life-time risk of developing globe rupture due to its weakened architecture (Ross et al., 2009).

Corneal cross-linking (CXL) is a Food and Drug Administration-approved, minimally invasive intervention that utilises ultraviolet A (UVA) and riboflavin (vitamin B_2_) to slow or even halt the progression of corneal ectasias. However, CXL is evolving. With our growing understanding of this procedure, modifications have been made not only to riboflavin’s formulation, but also to its delivery method and UV-A irradiation regime. Multiple assistive devices and adjunct procedures have also been designed to enhance the pharmacological effectiveness of riboflavin and UVA in inducing cross-links. This review aims to showcase the strides made to optimise and adapt CXL protocols according to the pharmacokinetic properties of riboflavin and UVA to *1*) improve upon their efficacy in treating corneal diseases, *2*) expand their utility to function as anti-infective therapy in the treatment of infective keratitis and, *3*) reduce toxicological profile in thin corneas.

### The Human Cornea and Its Effect on Ultraviolet Irradiation and Oxygen Consumption

The human cornea is an optically important structure. Its transparency enables passage of light into the retina while functioning primarily as a refractory surface, contributing approximately 70% of the eye’s total refractive power. Anatomically, the cornea is made up of six distinct layers - the epithelium, Bowman’s membrane, stroma, Dua’s layer, Descemet’s membrane, and the endothelium. The epithelial cells contain tight junction complexes to prevent ingress of paracellular fluid. Together with Bowman’s layer, these structures absorb UV radiation. The stroma contains a mixture of type I and V collagen fibrils oriented in a regular fashion, accounting for 90% of the entire cornea’s thickness. These interwoven fibrils are uniformly spaced by proteoglycans, forming stacked lamellae (Abahussin et al., 2009). Biomechanically, this arrangement confers viscoelasticity, protecting the cornea against deformation from environmental and intraocular pressures.

Besides its innate biomechanical structure, the cornea also contains an active fluid transport system to maintain its structural integrity. To support this metabolic system, the cornea requires a constant supply of oxygen. As an avascular structure (except for a limited peripheral zone), the cornea respires primarily across its anterior and posterior surfaces. Additionally, various components of the cornea consume varying amounts of oxygen. The epithelium utilises 40% of the total oxygen consumption of the cornea, while the stroma and endothelium consume 21% and 39% respectively. However, the per unit area of oxygen consumption of the epithelium is 10 times that of the stroma, and approximately 0.2 times that of the endothelium (Freeman, 1972).

## Mechanisms of Action

**FIGURE 1:**
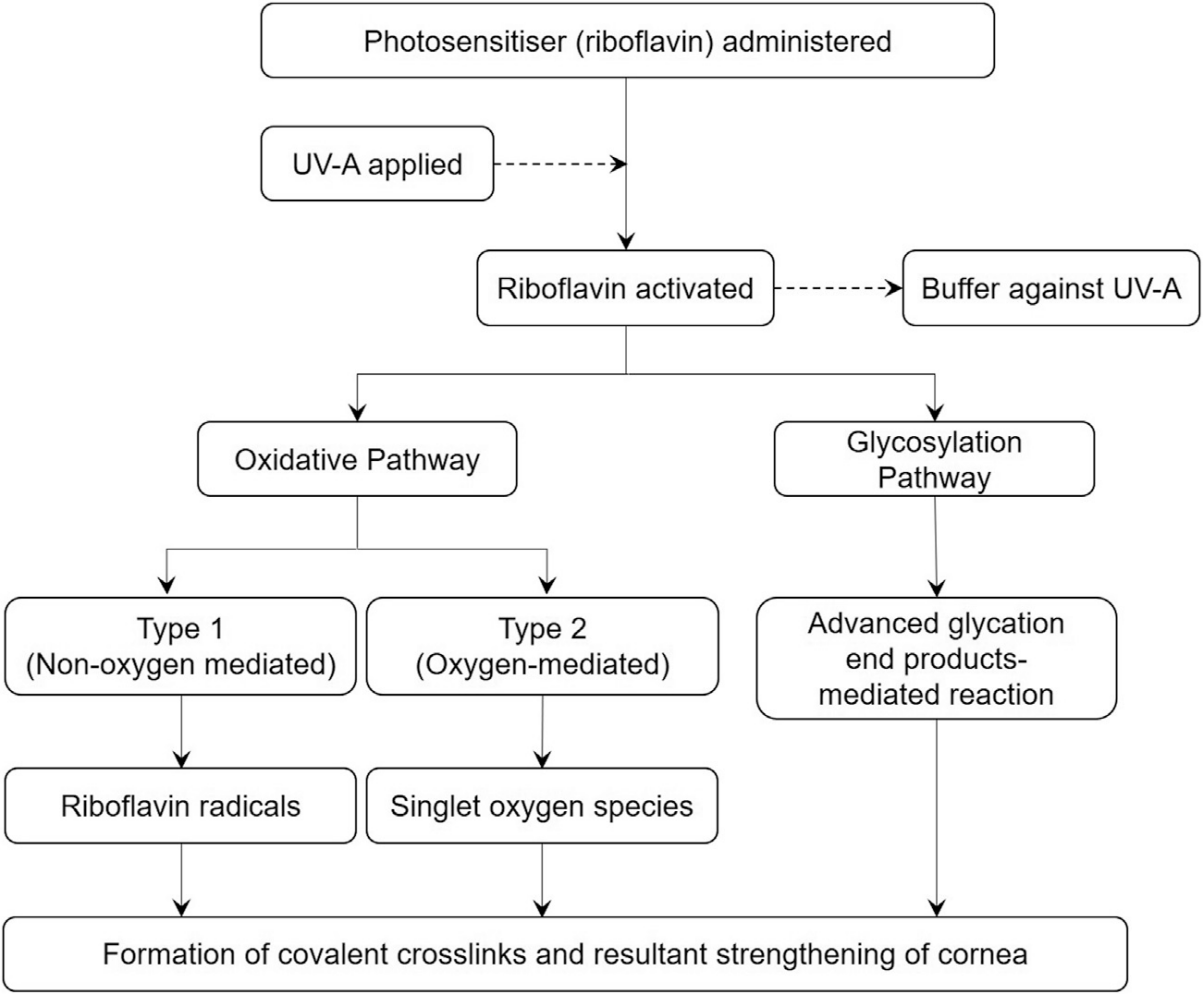
Proposed mechanisms of action of riboflavin.

The 2015 Global Consensus on Keratoconus and Ectatic Diseases highlighted riboflavin and UVA’s integral role in the treatment of corneal ectasias through strengthening of the corneal stroma (Gomes et al., 2015). At the molecular level, cross-links between corneal collagen monomers can be formed through enzymatic, glycosylation or oxidative pathways. Enzymatic cross-linking reactions from lysyl oxidase occur as part of the natural ageing process while glycosylation commonly occurs in patients with diabetes mellitus as part of the Maillard reaction (Dawczynski et al., 2002). These processes may explain reduced rates of keratoconus progression among people with diabetes and those older than 40-years-old. In corneal cross-linking, UVA and riboflavin promote cross-linking through both the oxidative and glycosylation pathways (Figure 1).

### Oxidative Pathway

A Type 1 (non-oxygen mediated) oxidative process occurs when riboflavin absorbs UV light, converting into an excited singlet riboflavin molecule that transforms into a triplet state (Raiskup and Spoerl, 2013). This reactive intermediate becomes a reservoir for hydride and electron transfer, creating covalent bonds between collagen fibrils and stromal substrates. A Type 2 (oxygen mediated) oxidative reaction occurs when UVA generates reactive oxygen species, which promotes formation of intra- and intermolecular covalent bonds to convert collagen monomers into cross-linked polymers. An understanding of these processes has been crucial in unveiling factors influencing the efficacy of CXL (Seiler et al., 2020).

### Glycosylation Pathway

Apart from oxidative reactions, riboflavin and UVA also promote glycosylative cross-linking within proteoglycans and collagen fibrils *via* advanced glycation end product-mediated mechanisms (Brummer et al., 2011). Additionally, riboflavin further functions as an optical buffer for UVA. It absorbs UVA, thereby reducing the risk of damage to posteriorly located structures such as the corneal endothelium, crystalline lens and retina (Wollensak et al., 2010).

## Dresden Protocol

Epithelium-off corneal cross-linking, or termed “Dresden protocol,” is the first widely recognised CXL protocol introduced by Dr. Theo Seiler and his team. This involves riboflavin 0.1% in 20% dextran and a UVA light source (365–370 nm) (Spoerl et al., 1998). Experimental animal studies have demonstrated increased cornea rigidity of between 42% and 70% in corneas treated with riboflavin and UVA (Seifert et al., 2014; Matteoli et al., 2016). Topical anaesthesia is administered to the ocular surface before debridement of the central 7–9 mm of corneal epithelium. Riboflavin is subsequently administered topically every 2 minutes for 30 minutes. Following which, irradiance of the central cornea of 3 mW/cm^2^, for a treatment duration of 30 minutes and surface dose of 5.4 J/cm^2^ was performed. Riboflavin is applied at 5 minute intervals throughout the treatment duration. Post-treatment, antibiotic eye drops are administered, and a bandage contact lens is placed over the cornea. Patients are instructed to administer topical antibiotics and steroids following this procedure.

Efficacy of the standard protocol in halting progression of keratectasia has been validated by multiple short-term and long-term studies. Current evidence shows that progression of keratoconus can be halted, with sustained improvements in clinical outcomes demonstrated for up to 10 years of follow-up (Raiskup and Spoerl, 2013; Wittig-Silva et al., 2014; Seyedian et al., 2015; Taşçı et al., 2020).

## Maximising Pharmacokinetic Properties of Riboflavin/Ultraviolet-A in the Treatment of Keratoconus

**FIGURE 2:**
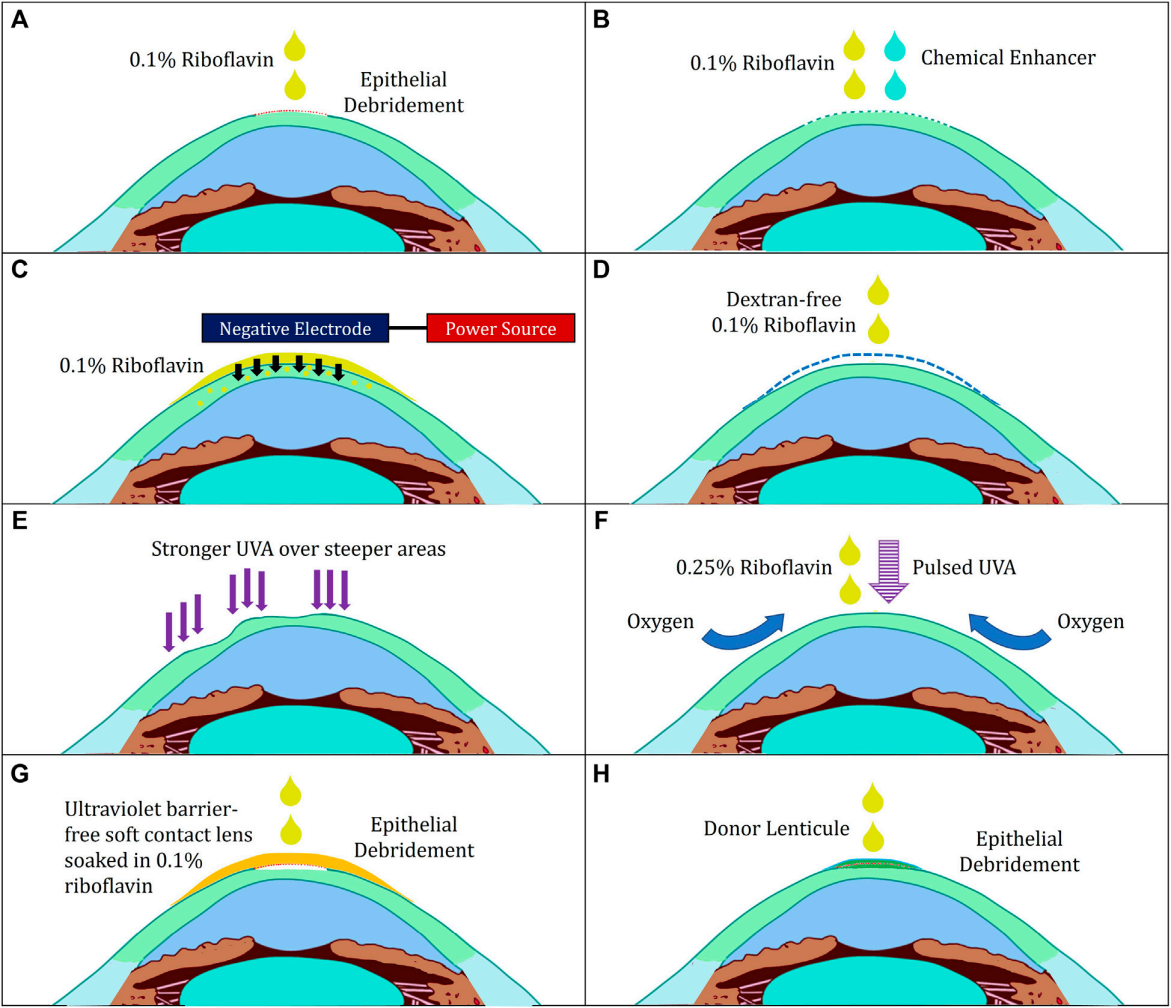
Illustration of various modifications to corneal cross-linking. **(A)** Standard “epithelium-off” Dresden Protocol **(B)** Transepithelial Protocol with Chemical Enhancers **(C)** Transepithelial Protocol with Iontophoresis **(D)** Hypoosmolar Riboflavin for Thin Corneas **(E)** Customised Protocol **(F)** Boost Epithelium-On Protocol **(G)** Contact-Lens Assisted Corneal Cross-Linking **(H)** Epi-Off-Lenticule-On Corneal Cross-linking.

To maximise treatment efficacy, safety and comfort, several modifications have been made according to the pharmacokinetic properties of riboflavin and UVA: *1*) UVA irradiation, *2*) riboflavin’s route of administration, *3*) oxygen supplementation and *4*) introduction of assistive modalities to improve visual acuity (Table 1; Figure 2).

**TABLE 1:** Summary of existing corneal cross-linking protocols.

Protocol	Riboflavin delivery	Ultraviolet-A	Oxygen delivery	Efficacy
Dresden protocol	Epithelium-off	3 mW/cm^2^; 30 min	Room air	Sustained clinical outcomes up to 10 years postoperatively. (Raiskup and Spoerl, 2013; Wittig-Silva et al., 2014; Seyedian et al., 2015; Taşçı et al., 2020)
Ultraviolet-A irradiation				
Accelerated protocol	Epithelium-off	Variable; 30 mW/cm^2^ for 3 min, 18 mW/cm^2^ for 5 min or 9 mW/cm^2^ for 10 minute	Room air	Regimens of low ultraviolet-A illumination with longer duration generally provide better results. (Peyman et al., 2016; Choi et al., 2017; Kirgiz et al., 2019) 9 mW/cm^2^ for 10 minute regimens are safe and efficacious in stabilizing keratoconus for up to 5 years follow-up (Mazzotta et al., 2021)
Ultraviolet-A-Emitting device	Epithelium-on 3 h/day for 6 months	0.31 mW/cm^2^ for 180 min daily for 6 months	Room air	*Ex-vivo* experiments on rabbit corneas have demonstrated that treated corneas with KeraVio were significantly stronger than the control group. Clinically, KeraVio halted corneal ectasia progression without any safety concerns in 20 eyes (Kobashi et al., 2020).
Riboflavin delivery				
Transepithelial protocol	Epithelium-on	3 mW/cm^2^; 30 min/variable	Room air	Less effective than Dresden protocol. (Soeters et al., 2015)
Transepithelial protocol with: (1) chemical enhancers	Epithelium-on; (1) Loosening of epithelial tight junctions	3 mW/cm^2^; 30 min/variable	Room air	Short term results comparable with Dresden protocol. (Wen et al., 2018).
(2) Iontophoresis	(2) electric field			
				More long-term comparative studies required. (Wen et al., 2018)
(3) Femto-second laser	(3) Femtosecond laser assisted			
Transepithelial protocol with phonophoresis	Epithelium-on	—	Room air	An experimental procedure demonstrated a statistically significant improvement in riboflavin penetration among ultrasound treated rabbit corneas (*p* < 0.001) (Lamy et al., 2013).
				However, hyperthermia is a potential safety concern of this technique.
Oral riboflavin	Epithelium-on	Exposure to 15 min of direct sunlight everyday	Room air	Series of three cases of keratoconus treated in this manner described no adverse effects and corneal flattening was reported within 6 months of treatment (Jarstad et al., 2019). A small prospective study is underway.
				Limited data is available regarding dose-response relationships of systemically absorbed riboflavin and its ocular bioavailability.
Improving oxygen diffusion				
Pulsed Ultraviolet-A	Epithelium-off/Variable	30 mW/cm^2^ for 4 min with a 1.5 s on/off cycle/Variable	Room Air	Stromal demarcation line was significantly deeper in the pulsed UVA group (213 ± 47.38 μm) compared to the continuous UVA group (149.32 ± 36.03 μm) (Moramarco et al., 2015).
				At 6 and 12 months, there was modest corneal flattening with keratometric stabilisation in 98.3% of eyes. No changes in central keratometry were noted. Moreover, mean corrected distance visual acuity, manifest refraction and endothelial cell density did not change (Gore et al., 2021)
Enhanced-fluence pulsed-light iontophoresis cross-linking	Epithelium-on with iontophoresis	18 mW/cm^2^ of pulsed-light on-off exposure	Room air	At 3 years, the average uncorrected distance visual acuity improved and average maximum keratometry readings decreased. Additionally, anterior segment optical coherence tomography showed that the demarcation lines were situated at an average depth of 285.8 ± 20.2 µm in more than 80% of patients at 1 month postoperatively, a value that is close to that of one created by standard epithelium-off cross-linking (Mazzotta et al., 2020b).
Periocular oxygen supplementation	Epithelium-on 0.25% riboflavin	10 J/cm^2^ (1 s: 1 s, pulsed)	Hyperoxic	Aydın *et al.* demonstrated that patients treated with periocular oxygen supplemented accelerated epithelium-on protocol experienced a larger decrease in maximum keratometry values (*p*-value = 0.019) and had a significantly deeper demarcation line (320 ± 17 µm) when compared to the control group (269 ± 19 µm) (Aydın and Aslan, 2021). Additionally, post-procedural endothelial cell density was comparable between both groups.
Optimising visual acuity				
Customised protocol (CurV)	Epithelium-off/Epithelium-on with oxygen supplementation	Customised according to corneal topography	Room air/Hyperoxic	One year results show stronger cornea flattening and faster healing time. (Seiler et al., 2016)
				Mazzotta *et al.* demonstrated that high-irradiance epithelium-on customised CXL with supplemental oxygen induces visual improvement and flattens steep keratometry. Additionally, demarcation lines were approximately 30% deeper in this series than previously reported by studies which utilised epithelium-off CurV protocols. This suggests the possibility of conducting CurV without the need for de-epithelisation (Mazzotta et al., 2020b).
				Long-term studies are warranted.
Athen’s protocol	Topographically-guided transepithelial photorefractive keratectomy (PRK) followed by corneal cross-linking	6 mW/cm^2^; 10 min	Room air	At 3 years Athen’s protocol offered superior uncorrected distance visual acuity and flatter steep and flat keratometry than the standard epithelium-off corneal cross-linking protocol. (Kymionis et al., 2009)
Cretan protocol	Transepithelial phototherapeutic keratectomy (tPTK) with corneal cross-linking	3 mW/cm^2^; 30 min	Room air	A 3 years prospective comparative study of 30 eyes demonstrated vision improvement and mean reduction in corneal astigmatism. In comparison, patients who underwent the standard epithelium-off protocol did not have any improvements in visual acuity or corneal astigmatism (Grentzelos et al., 2019).
Intrastromal corneal ring segment implantation with corneal cross-linking	Intrastromal corneal ring segment implantation with corneal cross-linking	9 mW/cm^2^; 10 min	Room air	A large prospective study of 542 eyes showed improvements in vision and maximum keratometry value in the CXL-ICRS group (Singal et al., 2020).
Anti-infective application				
PACK-CXL	Epithelium-off	3 mW/cm^2^; 30 min	Room air	Offered superior efficacy and healing duration in treating bacterial keratitis compared to antibiotics alone. (Tawfeek et al., 2020)
				Has a higher rate of corneal and worse visual acuity as compared to anti-fungals when treating fungal keratitis. Longer term studies are warranted. (Uddaraju et al., 2015; Prajna et al., 2020)
Thin corneas				
Hypoosmolar riboflavin	Dextran-free riboflavin solution	3 mW/cm^2^; 30 min	Room air	Stabilised keratectasia with no resulting endothelial cell loss (Hafezi et al., 2009; Raiskup and Spoerl, 2011)
Contact-lens assisted corneal cross-linking	Iso-osmolar riboflavin 0.1% Epithelium-off	3 mW/cm^2^; 30 min	Room air	Achieved a stromal demarcation line with mean depth of 252.9 ± 40.8 μm. No significant endothelial loss secondary to UVA toxicity was identified (Jacob et al., 2014).
				However, the presence of a contact lens over the epithelium creates an artificial barrier that reduces oxygen diffusion into the stroma (Kling et al., 2017; Wollensak et al., 2019).
Epithelial island cross-linking technique	Customised pachymetry guided epithelial debridement	3 mW/cm^2^; 30 min	Room air	Technique performed on 19 eyes with improvement in vision, flattest keratometry and steepest keratometry values reported 1 year postoperatively. However, there was significant endothelial cell density loss (2550 ± 324 vs 2030 ± 200 cells/mm^2^) 1 year postoperatively (Cagil et al., 2017).
Epi-off-lenticule-on corneal cross-linking	Epithelium-off	3 mW/cm^2^; 30 min	Room air	A recent study of this technique showed that visual acuity and endothelial cell density remained stable over a 12 months follow-up. All patients were observed to have a demarcation line by 6 months follow-up (Cagini et al., 2020).
Pachymetry-based accelerated cross-linking	Various based on the nomogram	Various based on the nomogram	Various	The M nomogram was validated against clinical findings of 20 eyes (Mazzotta et al., 2018).
				However, this protocol has a distinct limitation - it requires surgeons to have access to various riboflavin formulations, and cross-linking devices that can output UVA energy at 3, 9, 15, and even 30 mW/cm^2^, using either continuous light or pulsed light protocols. Moreover, iontophoresis may even be required in some cases to perform the treatment.
Sub400 protocol	Epithelium-off	UV illumination time and irradiance adjusted according to the corneal thickness to achieve a safe depth of cross-linking 70 µm away from the endothelium	Room air	Pilot study showed that 90% of 39 thin corneas ranging from 214 to 398 µm achieved topographical stability at 12 months, and no eyes experienced endothelial decompensation as a result of UV irradiation toxicity. (Hafezi et al., 2020)
Other indications				
LASIK Xtra and SMILE Xtra	Concomitantly with LASIK/SMILE	3 mW/cm^2^; 30 min	Room air	Variable short-term results reported. Longer termed and larger scale studies required. (Kanellopoulos and Asimellis, 2015; Konstantopoulos et al., 2019; Kohnen et al., 2020)
Hyper-osmolar riboflavin	Preoperative 40% glucose or intraoperative 70% glycerol	3 mW/cm^2^; 30 min	Room air	Reduction of central corneal thickness and visual acuity observed in patients with bullous keratopathy. (Wollensak et al., 2009; Hafezi et al., 2010)

### UVA Irradiation

UVA interacts with riboflavin to activate oxidative and glycosylation pathways that lead to the formation of collagen cross-links. A dosage (fluence) of 5.4 J/cm^2^ is required to achieve the desired cornea stiffening. The original Dresden protocol utilises UVA irradiation at 3 mW/cm^2^ for a treatment duration of 30 minutes. In a bid to improve patient comfort, “accelerated” protocols have been proposed.

#### Accelerated Protocols

“Accelerated” protocols have been developed to deliver higher UVA irradiance at a shorter duration to reduce patient discomfort. Several variations in UVA exposure have been described to achieve a cumulative dose of 5.4 J/cm^2^: 30 mW/cm^2^ for 3 minutes, 18 mW/cm^2^ for 5 minutes or 9 mW/cm^2^ for 10 minutes. This is based on Bunsen-Roscoe’s law of photochemical reciprocity, which describes a linearly proportional photochemical effect to the total UV energy delivered, regardless of duration of administration (Schindl et al., 2001). A prospective non-randomised interventional study of 156 eyes with early progressive keratoconus demonstrated that accelerated epithelium-off CXL with UVA irradiation of 9 mW/cm^2^ for 10 minutes was associated with favorable outcomes (Mazzotta et al., 2021). The 5 years results demonstrated sustained improvements in uncorrected distance visual acuity, corrected distance visual acuity and maximum keratometry values. A mean demarcation line depth of 332.6 ± 33.6 μm was identified on anterior segment optical coherence tomography. Although the presence of corneal haze was noted in 11.6% of patients, this resolved in all patients following initiation of topical steroid therapy (Mazzotta et al., 2021). This study demonstrates the long-term efficacy and safety of 9 mW/cm^2^ accelerated CXL protocols, providing evidence supporting the use of accelerated CXL protocols in the treatment of early keratoconus.

Two other prospective studies also showed keratoconus stabilisation with improvements in visual acuity after patients received accelerated CXL (Mimouni et al., 2021; Sot et al., 2021). Interestingly, Mimouni *et al.* reported that patients with central cones (defined as within the central 3 mm of the cornea) demonstrated greater improvement in best spectacle-corrected visual acuity (0.08 ± 0.02 logMAR, *p* < 0.001) and myopia (1.27D more reduction) than patients with paracentral cones (within 3–5 mm of the central cornea) (Mimouni et al., 2021). Sot *et al.*, on the other hand, reported that 17.1% of patients in a cohort of 82 eyes treated with accelerated CXL showed signs of progression. These patients were observed to be younger, with higher maximum keratometry readings and more pronounced optical aberrations (Sot et al., 2021).

A meta-analysis comparing accelerated vs. conventional epithelium-off protocols reported stabilisation of keratometry values up to 1 year after the procedure in both groups, with no statistical difference in maximum keratometry values at 1 year of follow-up. Additionally, no statistical differences in post-procedure endothelial cell density were noted between groups. However, patients treated with the Dresden protocol exhibited better corrected visual acuity at 1 year follow-up and had a deeper demarcation line than those treated with an accelerated protocol (Kobashi and Tsubota, 2020).

A multi-centre retrospective study which included 684 eyes, examined patients undergoing both interventions and demonstrated similar outcomes (Kandel et al., 2021). Both protocols halted progression (defined as less than a dioptre increase in maximum keratometry) with similar efficacy (accelerated: 89% vs conventional: 88%). Patients in the Dresden protocol group experienced a greater improvement in adjusted mean pinhole visual acuity (4.4 vs 1.6 logMAR, *p*-value = 0.04). Moreover, a higher proportion of patients in the accelerated group experienced clinically significant corneal haze (17.7 vs 10.2%, *p*-value = 0.02). While accelerated protocols provide the advantage of reducing surgical time without jeopardising short-term disease stabilisation, further studies regarding long-term outcomes are required.

Accelerated protocols commonly utilise UVA irradiance ranging between 9 mW/cm^2^ and 30 mW/cm^2^. However, previous clinical studies have shown that regimens of low UVA illumination with longer duration induces more cross-links with riboflavin, implying that Bunsen-Roscoe’s law does not adequately predict the efficacy of accelerated UVA regimens (Peyman et al., 2016; Choi et al., 2017; Kirgiz et al., 2019). This observation had been further supported by *ex-vivo* studies, demonstrating that UV fluence is non-linear for UVA illumination intensities above 9 mW/cm^2^ (Wernli et al., 2013; Hammer et al., 2014; Lin and Cheng, 2017). Therefore, accelerated CXL protocols may have varying efficacy rates, dependent upon the UVA regimen used. Although current evidence suggests that lower irradiance produces more cross-links, the optimal UVA regimen required to halt keratoconus progression is still not known (Seiler et al., 2020).

This observation most likely arises from reduced oxygen availability, which is crucial for the CXL reaction and its resultant biomechanical stiffening (Richoz et al., 2013). Demonstration of a significant reduction in oxygen availability during CXL at higher UVA irradiances may explain the reduced strengthening effect of accelerated protocols; where short illumination duration with high UVA intensities provides decreased time for oxygen re-diffusion to occur to produce oxygen-mediated cross-links (Seiler et al., 2020). Protocols such as the Boost Epi-On Protocol have been formulated to address these observations.

#### Ultraviolet-A-Emitting Device

The Dresden protocol requires patients to be stationary for an extended duration. KeraVio is a CXL treatment modality which utilises UVA emitting glasses and topical epithelium-on riboflavin administration for 3 h daily across 6 months. This allows patients to be ambulatory, thereby reducing patient discomfort during the procedure. *Ex-vivo* experiments on rabbit corneas have demonstrated that treated corneas with KeraVio were significantly stronger than controls (Kobashi et al., 2020). Clinically, KeraVio halted corneal ectasia progression without any safety concerns in 20 eyes (Kobashi et al., 2020). Further comparative studies are required to ascertain the efficacy of this technique.

### Riboflavin Delivery

#### Epithelium-On Protocol

Riboflavin is a large hydrophilic molecule that does not penetrate tight junctions of the intact corneal epithelium (Gore et al., 2015). The Dresden protocol overcame this through epithelial debridement (“epithelium-off”), allowing diffusion of riboflavin molecules into the stroma. Even though epithelial debridement provides favourable riboflavin penetration, this is associated with risks such as pain, infection, persistent epithelial defects, and corneal melt (Spoerl et al., 2007; Evangelista and Hatch, 2018).

To mitigate these problems, epithelium-on protocols, where riboflavin is administered directly on an intact corneal epithelium, were devised. Although epithelium preservation increases corneal thickness and may confer protection to the underlying endothelial cells, its barrier function greatly limits the extent of riboflavin absorption, UVA penetration and oxygen availability. Consequently, epithelium-on protocols failed to demonstrate satisfactory clinical efficacy. A randomised-controlled trial reported evidence of keratoconus progression in 23% of eyes undergoing epithelium-on corneal cross-linking, while progression was halted in all epithelium-off eyes (Soeters et al., 2015). Meta-analyses performed by Li and Wang, and Nath *et al.* confirmed that epithelium-on protocols were less effective. Li and Wang demonstrated that standard epithelium-off protocols were more effective at reducing maximum corneal curvature than epithelium-on protocols (Li and Wang, 2017), while Nath *et al.* reported that 7% of patients undergoing epithelium-on CXL experienced disease progression within the first year, compared to 2% of patients in the epithelium-off group (*p*-value = 0.022) (Nath et al., 2020). As a result, several modifications to epithelium-on CXL have been proposed.

#### Chemical Enhancers

Topical medications such as tetracaine, benzalkonium chloride, ethylenediaminetetraacetic acid and trometamol, which are toxic to the corneal epithelium, have been used to increase permeability of tight intraepithelial junctions and promote riboflavin diffusion through the epithelium (Cha et al., 2004). Meta-analysis of eight short-term 1 year studies of 455 keratoconus eyes showed that eyes treated with epithelium-on protocols and chemical enhancers experienced a comparable reduction in corneal curvature as eyes treated with standard epithelium-off protocol (Wen et al., 2018).

However, long-term results suggest that epithelium-on protocols with chemical enhancers alone are less effective compared to epithelium-off protocols. A 3 year comparative study of an epithelium-on protocol with chemical enhancers vs. standard epithelium-off patients established that although keratoconus progression was halted in both groups, epithelium-off patients demonstrated superior corneal aberrometry and asphericity results (Arance-Gil et al., 2020).

#### Iontophoresis-Assisted Riboflavin Delivery

Iontophoresis has also been studied as an adjunct to improve riboflavin penetration in epithelium-on protocols. In iontophoresis-CXL, an electric field is created to increase diffusion of negatively charged riboflavin through the epithelium and stroma. A meta-analysis of 455 eyes with keratoconus by Wen *et al.* found that although comparable reduction in corneal curvature in eyes treated with standard epithelium-off protocol was achieved, patients treated with iontophoresis-assisted transepithelial protocols experienced lower reduction in corneal curvature compared to those treated with standard epithelium-off protocols (Wen et al., 2018).

#### Phonophoresis-Assisted Riboflavin Delivery

It is postulated that phonophoresis enhances pharmacological delivery *via* radiation forces, acoustic streaming, and acoustic cavitation (Pitt et al., 2004). An experimental procedure by Lamy *et al.* involved the use of ultrasound to augment the penetration of riboflavin into the corneal stroma over an intact epithelium. In this study, the authors utilised fluorescent riboflavin and an ultrasound device adjusted to produce continuous-wave ultrasound of 880 kHz at 1 W/cm^2^ to aid delivery of the drug. A statistically significant difference between riboflavin penetration was noted between non-ultrasound treated rabbit corneas and ultrasound treated rabbit corneas (*p* < 0.001). The authors concluded that ultrasound treatment aided entry of riboflavin into the corneal stroma despite the presence of an intact epithelial barrier (Lamy et al., 2013). At the same time, a mean increase in temperature of 6–7°C in eyes undergoing phonophoresis-assisted riboflavin delivery was identified. This raises safety concerns, as hyperthermia of 41°C and beyond has been associated with the development of cataracts and elevated corneal epithelium 70-kilodalton stress protein (Nabili et al., 2015). Further clinical studies are needed to further characterise the safety aspects of this method.

#### Oral Riboflavin

A novel approach to CXL without the need for epithelial debridement involves administration of oral riboflavin and natural sunlight exposure. In this proposed technique, patients ingest high doses of riboflavin and are exposed to 15 minutes of direct sunlight while engaging in daily exercise. A series of three cases of keratoconus treated in this manner described no adverse effects, with corneal flattening reported within 6 months of treatment (Jarstad et al., 2019). A small prospective study of 24 patients with keratoconus is underway to investigate the efficacy of high dose (400 mg) oral riboflavin (Jarstad et al., 2019).

Although oral riboflavin presents itself as an inexpensive and less invasive alternative to corneal cross-linking, its utility may be limited by the extended duration of treatment, variability in ultraviolet exposure and patient compliance to outdoor regimens. Additionally, given the long treatment duration and hence slower formation of cross-links, this method may not be optimal for patients with severe progressive keratoconus, which requires rapid stabilisation to prevent further progression. Limited data is also available regarding dose-response relationships of systemically absorbed riboflavin and its ocular bioavailability. Furthermore, the toxicity of systemic administration of high doses of riboflavin has yet to be well-established (Institute of Medicine US, 1998). The systemic administration of riboflavin may also function as an endogenous photosensitiser, which confers an increased risk of experiencing sunburn and photoaging of the skin (Pandel et al., 2013).

### Improving Oxygen Diffusion

Oxygen is a key component of the Type 2 oxidative pathway in the reaction between riboflavin and UVA. However, in epithelium-on protocols, stromal oxygen diffusion is limited by the intact epithelium. The corneal epithelium absorbs almost ten times the amount of oxygen that the corneal stroma does (Freeman, 1972). Therefore, riboflavin’s cross-linking efficacy in epithelium-on protocols is reduced within an oxygen-poor stromal environment, (Richoz et al., 2013). To overcome this, several solutions have been proposed.

#### Pulsed Ultraviolet-A

Previous studies have shown that continuous high UVA irradiation results in unsatisfactory riboflavin-induced corneal stiffening due to inadequate oxygen diffusion (Peyman et al., 2016; Choi et al., 2017; Kirgiz et al., 2019). Hence, it was suggested that pulsed fractionation of UVA irradiation may improve cross-linking efficacy by allowing re-diffusion of oxygen during pauses in between UVA light pulses (Richoz et al., 2013).

This postulation was confirmed by a randomised controlled trial of 60 patients conducted by Moramarco *et al.* which compared accelerated CXL using continuous UVA exposure at 30 mW/cm^2^ for 4 minutes with accelerated CXL using pulsed UVA with 8 minutes (1 second on: 1 second off) of UVA exposure at 30 mW/cm^2^. Their study showed that the stromal demarcation line was significantly deeper in the pulsed UVA group (213 ± 47.38 μm) compared to the continuous UVA group (149.32 ± 36.03 μm) (Moramarco et al., 2015). Another similar randomised controlled trial of 70 eyes conducted by Peyman *et al.* corroborated these findings, with a significantly deeper stromal demarcation line observed in the pulsed group compared to the continuous group (201.11 ± 27.76 vs 159.88 ± 20.86 µm) (Peyman et al., 2016). However, under laboratory settings, pulsed ultraviolet-A light alone did not substantially improve the increase in corneal biomechanical strength (Kling et al., 2015).

Gore *et al.* studied an accelerated pulsed high-fluence protocol for progressive keratoconus on 756 eyes (Gore et al., 2021). Corneas with thickness <375 μm were excluded. High-fluence, pulsed ultraviolet-A was delivered at 30 mW/cm^2^ for 4 minutes with a 1.5 s on/off cycle. The total energy delivered was 7.2 J/cm^2^. At 6 and 12 months, the study team noted modest corneal flattening with keratometric stabilisation in 98.3% of eyes. No changes in central keratometry were noted. Moreover, mean corrected distance visual acuity, manifest refraction and endothelial cell density did not change.

#### Enhanced-Fluence Pulsed-Light Iontophoresis Cross-Linking

Due to its improved efficacy, pulsed UVA irradiation was studied as an adjunct to the iontophoresis-assisted epithelium-on protocol to improve cross-linking efficiency. Mazzotta *et al.* conducted a prospective interventional pilot study of 24 eyes of 20 patients with keratoconus. Patients underwent iontophoresis-assisted cross-linking with riboflavin solution and received UVA irradiation of 18 mW/cm^2^ of pulsed-light on-off exposure. This combination of increased riboflavin transport and oxygen availability across the epithelium produced favourable results.

At 3 years, the average uncorrected distance visual acuity improved from 0.50 ± 0.10 to 0.36 ± 0.08 logMAR and average maximum keratometry readings decreased from 52.94 ± 1.34 to 51.4 ± 1.49 diopters. Additionally, anterior segment optical coherence tomography showed that the demarcation lines were situated at an average depth of 285.8 ± 20.2 µm in more than 80% of patients at 1 month postoperatively (Mazzotta et al., 2020a). This shows that with pulsed UVA irradiation, epithelium-on cross-linking can achieve a demarcation line depth that is close to that of one created by the standard epithelium-off cross-linking. Further comparative clinical studies with epithelium-off protocols are warranted.

#### Oxygen Supplementation

Besides pulsed UVA, oxygen supplementation of the corneal surface has been shown to increase the strength and depth of CXL (Seiler et al., 2020). A wearable oxygen delivery device, known as Boost (Avedro, MA, United States), is a recent approach to epithelium-on corneal cross-linking. It creates a hyperoxic periocular environment. *Ex-vivo* studies have shown that periocular oxygen supplementation provides significantly more corneal stiffening (Adler et al., 2019). A randomised, age-sex-matched study by Aydın *et al.* demonstrated that patients treated with periocular oxygen supplemented accelerated epithelium-on protocol experienced a larger decrease in maximum keratometry values (*p*-value = 0.019) and had a significantly deeper demarcation line (320 ± 17 µm) when compared to the control group (269 ± 19 µm) (Aydın and Aslan, 2021). Additionally, post-procedural endothelial cell density was comparable between both groups.

### Improving Visual Acuity

Although epithelium-off riboflavin and UVA cross-linking is highly effective in halting the progression of keratoconus, it provides limited improvement in visual acuity (Raiskup and Spoerl, 2013; Wittig-Silva et al., 2014; Seyedian et al., 2015; Taşçı et al., 2020). As a result, several adjunct devices and procedures have been used in combination with riboflavin administration to improve visual outcomes.

#### Photorefractive Keratectomy With Corneal Cross-Linking

The Athens protocol involves performing topographically-guided transepithelial photorefractive keratectomy (PRK) followed by CXL. Early studies have shown that a combination of PRK with CXL offers keratoconic eyes better visual acuity and corneal stability (Kymionis et al., 2009). A recent study by Kontadakis *et al.* compared simultaneous topography-guided PRK and CXL (tPRK-CXL group) with CXL alone and found that uncorrected distance visual acuity at 3 years was significantly better in the tPRK-CXL group (0.27 ± 0.25 logMAR) as compared to the CXL-only group (0.69 ± 0.58 logMAR). Moreover, steep and flat keratometric readings were also flatter in the tPRK-CXL group (Kontadakis et al., 2016).

Kanellopoulos *et al.* further proposed the enhanced Athens protocol, incorporating a customised and fluence topography-guided UVA irradiation to maximise refractive normalisation of the cornea with lesser stromal tissue removal than the standard Athens protocol (Kanellopoulos, 2019). Finally, Minneapolis’ epi-off protocol, which utilises simultaneous CXL with Phorcides analytical software topography-guided correction photorefractive keratectomy is also being explored as a treatment modality (Hammond and Lobanoff, 2019).

#### Transepithelial Phototherapeutic Keratectomy With Corneal Cross-Linking

The Cretan protocol involves performing transepithelial phototherapeutic keratectomy (tPTK) with corneal cross-linking. In 2010, Kymionis *et al.* reported a case of keratoconus treated with the Cretan protocol. This patient achieved an improvement in postoperative uncorrected visual acuity and best spectacle-corrected visual acuity with stabilisation of keratoconus progression (Kymionis et al., 2010). A clinical comparative study of the Cretan and Dresden protocol subsequently showed that the mean uncorrected distance visual acuity and corrected distance visual acuity of eyes treated with the Cretan protocol improved from logMAR 0.99 ± 0.71 and 0.30 ± 0.26 preoperatively to 0.63 ± 0.42 and 0.19 ± 0.18 at 12 months postoperatively, respectively. Neither uncorrected nor corrected distance visual acuity demonstrated any significant improvement postoperatively, and at 12 months in the Dresden protocol group (Kymionis et al., 2012a). A 3 year prospective comparative study of 30 eyes conducted by Grentzelos *et al.* showed that not only did uncorrected and corrected distance visual acuity improve up to 3 year postoperatively, mean corneal astigmatism was also reduced from −6.19 ± 4.54 diopters preoperatively to −4.68 ± 3.10 diopters. Patients who underwent routine epithelium-off CXL did not experience improvements in visual acuity or corneal astigmatism (Grentzelos et al., 2019).

#### Intrastromal Corneal Ring Segment Implantation With Corneal Cross-Linking

Intrastromal corneal ring segment implantation has been studied as a treatment modality for corneal ectasias. Alió *et al.* studied the effects of intrastromal corneal ring segment implantation in eyes with keratoconus, while Kymionis *et al.* followed up on eyes with post-LASIK ectasia (Alió et al., 2006; Kymionis et al., 2006). Both studies showed that long-term refractive stability was achieved. However, keratometry values worsened after 36 months amongst eyes with keratoconus (Alió et al., 2006). Kim *et al.* subsequently reported that intracorneal ring segment implantation followed by CXL with riboflavin within 1 month had a greater effect on improvements in visual acuity and reduction in refractive and keratometric values compared to intracorneal ring segment implantation or CXL alone (Kim and Kim, 2019).

Singal *et al.* conducted a large prospective study of 542 eyes comparing CXL alone (*n* = 204) (CXL-alone), CXL with intracorneal ring segment implantation (*n* = 126) (CXL-ICRS) and topography-guided photorefractive keratectomy with CXL (*n* = 122) (CXL-TG-PRK) in patients with progressive keratoconus, pellucid marginal degeneration, or LASIK-induced ectasia. It was noted that changes in uncorrected distance visual acuity was significant in patients undergoing CXL-ICRS (−0.31; 95% CI, −0.38 to −0.24) and CXL-TG-PRK (−0.16; 95% CI, −0.24 to −0.09), but not in CXL-only. Moreover, changes in maximum keratometry value were significant amongst eyes which underwent CXL-ICRS (−3.21 diopters (D); 95% CI, −3.98 to −2.45) and CXL-TG-PRK (−3.69 D; 95% CI, −4.49 to −2.90), but not with CXL alone (−0.05 D; 95% CI, −0.66 to 0.55) (Singal et al., 2020). The authors concluded that CXL with intrastromal corneal ring segment implantation may be more effective for eyes with greater irregular astigmatism and worse visual acuity, while topography-guided photorefractive keratectomy with CXL is effective for eyes requiring improvement in irregular astigmatism but still with good corrected distance visual acuity.

#### Customised Remodelled Vision (CurV) Protocol

Excitingly, the new Customised Remodelled Vision (CurV) protocol can improve the quality of vision by individualising UVA irradiation patterns guided by corneal topography (Figure 2). It is theorised that corneal biomechanical weakness in keratoconus is focal in nature (Roberts and Dupps, 2014). Hence, in contrast to the generalised UVA irradiation administered with the Dresden protocol, CurV irradiates ectatic parts of the cornea with more intense UVA irradiation whilst stronger areas are treated with little or no UVA. One year results by Seiler *et al.* were encouraging, with patients treated with CurV showing greater changes in maximum keratometry values (−1.7 ± 2.0 diopter vs. −0.9 ± 1.3 diopter) and superior epithelial healing time (2.56 ± 0.50 days vs. 3.19 ± 0.73 days) than patients treated with an epithelium-off protocol (Seiler et al., 2016). These findings are further supported by a study performed by Cassagne *et al.*, who found that CurV induced a greater change in maximum keratometry values compared to standard epithelium-off protocol and improved corrected distance visual acuity (0.2162 ± 0.2495 logMAR vs 0.2648 ± 0.2574 logMAR) over a follow-up duration of a year (Cassagne et al., 2017).

Mazzotta *et al.* recently investigated the utility of high-irradiance epithelium-on customised CXL with supplemental oxygen (Mazzotta et al., 2020b). A total of 27 eyes were included in this study. Patients experienced significant improvement in corrected distance visual acuity with flattening of steep keratometry (mean change of −1.9 dioptres, *p*-value<0.05) at their follow-up 6 months after the procedure. Additionally, two demarcation lines were observed at mean depths of 218.23 ± 43.32 µm and 325.71 ± 39.70 µm. Notably, these demarcation lines were approximately 30% deeper in this series than previously reported by studies which utilised epithelium-off CurV protocols. This suggests the possibility of conducting CurV without the need for de-epithelisation. More long-term comparative studies are warranted to determine the efficacy of CurV.

## Anti-Infective Application of Riboflavin and Ultraviolet-A for the Treatment of Infective Keratitis (PACK-CXL)

CXL has been studied as an alternative to antibiotics in the treatment of infectious keratitis. Termed photoactivated chromophore for infectious keratitis - corneal cross-linking (PACK-CXL), it has been suggested to provide added resistance to pepsin and collagenase enzymes produced by bacterial and fungal pathogens (Hafezi and Randleman, 2014; Vajpayee et al., 2015). Additionally, UVA and riboflavin have been postulated to confer synergistic antimicrobial effects. UVA damages microbial DNA and RNA; thereby inhibiting microbial replication, while photoactivated riboflavin releases free radicals that damage cell membranes and nucleic acids (Vazirani and Vaddavalli, 2013).

### Bacterial Keratitis

Recent studies have shown that PACK-CXL offers superior efficacy and reduces duration of healing of bacterial keratitis. Tawfeek *et al.* randomised 78 eyes with bacterial keratitis into a control group receiving appropriate topical conventional broad-spectrum antimicrobial therapy alone and a group with topical antibiotics with adjuvant PACK-CXL. It was noted that a higher proportion of patients in the group receiving combination treatment of antibiotics with PACK-CXL experienced complete resolution of ulcers (97.4 vs 76.9%, *p*-value<0.001). Additionally, the mean resolution period was shorter in patients treated with PACK-CXL (7.02 ± 2 weeks vs 10.87 ± 3.28 weeks, *p*-value = 0.002) (Tawfeek et al., 2020).

Knyazer and colleagues have reported that additional PACK-CXL significantly reduces time to epithelial healing in ulcers up to 4 mm in size when using accelerated PACK-CXL and standard-fluence of 5.4 J/cm^2^ (Knyazer et al., 2020). A recently performed prospective randomized Swiss multicenter PACK-CXL trial further demonstrated that PACK-CXL alone is as efficient as antimicrobial therapy in small ulcers up to 4 mm in size (Torres-Netto et al., 2020).

In patients with treatment-resistant post-penetrating keratoplasty infectious keratitis, PACK-CXL in comparison to medical therapy alone, offered greater rates of resolution (83.3 vs 68.2%, *p*-value = 0.28), healing duration (average of 24 vs 34 days, *p*-value = 0.02) and reduction in graft failure rates (27.8 vs 54.5%, *p*-value = 0.08) in the PACK-CXL group, although some of these results were not statistically significant (Ozbek-Uzman et al., 2020).

### Fungal Keratitis

In contrast, PACK-CXL has been reported to be associated with increased risk of corneal perforation and visual acuity deterioration when used to treat fungal keratitis (Uddaraju et al., 2015; Prajna et al., 2020). A randomised control trial of 111 patients found no difference in culture positivity at 24 hours, re-epithelisation rates or scar size between those randomised to anti-fungals alone compared to PACK-CXL with antifungals (Prajna et al., 2020). Moreover, the best spectacle-corrected visual acuity at 3 weeks and 3 months appeared to deteriorate in the PACK-CXL group. However, concerns were raised from two different groups regarding the methodology of the study (Ting et al., 2020; Hafezi et al., 2021b). On the other hand, Wei *et* al. randomized 41 patients with fungal keratitis into two groups–one group was treated both CXL and antifungals, while patients in the other group were treated with antifungals only. They demonstrated that CXL in combination with antifungals accelerated the duration of ulcer healing, reduced the frequency of administered medications, and significantly reduced the maximum ulcer depth after treatment (Wei et al., 2019). Rose Bengal Photodynamic Antimicrobial Therapy (RB-PDAT) has been studied as an alternative, and a pilot study of 18 eyes showed that 72% of the treated eyes avoided the need for therapeutic penetrating keratoplasty (Naranjo et al., 2019).

## Reducing Riboflavin/Ultraviolet-A Toxicity for the Treatment of Thin Corneas

### Toxicology Profile of Riboflavin and Ultraviolet-A

Ultraviolet rays have the propensity to induce structural damage depending on its wavelength, intensity, and irradiation duration. Direct exposure to ultraviolet rays may induce photokeratitis, endothelial damage, cataracts, and even retinal injury. However, ultraviolet-A-induced damage is unlikely to occur in CXL because riboflavin shields these structures from potential injury (Spoerl et al., 2007). The degree of ultraviolet-A shielding by riboflavin is believed to be governed by the Beer-Lambart Law, which states a linear relationship between the concentration and absorbance of the solution (riboflavin) (Spoerl et al., 2007; Iseli et al., 2011). Experimentally, this relationship holds true for lower concentrations of riboflavin - the absorbance coefficient of riboflavin solutions increases linearly up to a concentration of 0.04%, before plateauing and remaining constant for higher concentrations (Spoerl et al., 2007). Hence, injury secondary to direct ultraviolet-A irradiation is unlikely to occur in a clinical setting where 0.1% riboflavin is used.

However, Wollensak *et al.* reported that photopolymerisation between riboflavin and ultraviolet-A produces cytotoxic free radicals which can cause significant damage, especially to the corneal keratocytes and endothelial cells (Wollensak et al., 2003b; 2004b; 2004a). Furthermore, Caporossi *et al.* showed that of 10 eyes treated with riboflavin/ultraviolet-A clinically, keratocyte apoptosis occurred up to depths ranging from 270 to 350 μm. Fortunately, such damage appears to be transient as repopulation of keratocytes with a normal keratocyte density within the entire corneal stroma was observed at 6 months (Caporossi et al., 2006).

Conversely, endothelial cell damage after CXL resulting in severe corneal oedema that impedes visual acuity have been reported in literature (Bagga et al., 2012; Sharma et al., 2012). Wollensak *et al.* demonstrated that ultraviolet-A irradiance of 0.35 mW/cm^2^ causes endothelial cell toxicity at depths greater than 400 μm (Wollensak et al., 2003b). Moreover, Kymionis *et al.* reported a significant decrease of endothelial cell density (mean decrease of 292 cells/mm^2^) after performing standard epithelium-off corneal crosslinking in 14 eyes with thin corneas (range 340–399 μm) (Kymionis et al., 2012b). A case series by Sharma *et al.* identified 10 patients out of a cohort of 350 patients with persistent post-CXL corneal oedema, which was postulated to have occurred from inadvertent corneal endothelial damage (Sharma et al., 2012). Therefore, a minimum corneal thickness of 400 μm was previously suggested as a prerequisite for epithelium-off CXL due to the risk of endothelial cell toxicity (Sharma et al., 2012)

Given the above-mentioned risk of endothelial cell toxicity, a potential challenge in CXL is treating patients with severe disease where their corneal thickness is less than 400 μm. To overcome this, multiple solutions such as altering the osmolarity of riboflavin formulation, using assistive techniques to change riboflavin delivery and customising UVA irradiation to induce riboflavin cross-link formations at varying depths have been proposed.

### Hypoosmolar Riboflavin

Hypoosmolar riboflavin in a dextran-free solution has been used to induce stromal swelling and consequently increase corneal thickness in patients with thin corneas. Corneal swelling occurs due to hydrophilic properties of stromal proteoglycans, which form “collagen-free lakes” and increases corneal thickness (Hafezi et al., 2009). Several studies have shown that hypoosmolar CXL formulations for thin corneas aids in the stabilisation of keratoconus with no resulting endothelial cell loss (Hafezi et al., 2009; Raiskup and Spoerl, 2011). However, the efficacy of hypoosmolar riboflavin has been suggested to be less pronounced compared with standard formulations (Stojanovic et al., 2014). This may occur due to reduced concentrations of collagen fibrils and poor oxygen diffusion secondary to increased hydration of the corneal stroma. Additionally, Hafezi *et al.* have reported that hypoosmolar riboflavin results in large interindividual variations in corneal swelling duration and range (swelling duration ranged from 3 to 20 minutes extent of swelling ranged from 36 to 105 μm) (Hafezi et al., 2009).

### Contact-Lens Assisted Corneal Cross-Linking (CACXL)

In 2014, Jacob *et al.* described a novel method of inducing corneal swelling in patients with keratoconus and thin corneas measuring between 350 μm and 400 μm. The proposed protocol involved placing an ultraviolet barrier-free soft contact lens (0.09-mm thickness, 14-mm diameter) soaked in iso-osmolar riboflavin 0.1% for 30 minutes on the cornea. Once the corneal thickness was confirmed to be greater than 400 μm, UVA irradiation was commenced combined with administration of iso-osmolar riboflavin 0.1%. Their study found that CACXL achieved a stromal demarcation line mean depth of 252.9 ± 40.8 μm, with no significant endothelial loss secondary to UVA toxicity identified (Jacob et al., 2014). However, the presence of a contact lens over the epithelium creates an artificial barrier that reduces oxygen diffusion into the stroma, which reduces efficacy of riboflavin and UVA in inducing cross-linking (Kling et al., 2017; Wollensak et al., 2019).

### Epithelial Island Cross-Linking Technique

Another potential treatment which adjusts the way riboflavin is applied to the corneal surface is customised pachymetry guided epithelial debridement. This technique requires epithelial debridement over areas of thicker cornea, while leaving an “island” of undebrided epithelium over the thinner apical area. Once riboflavin is applied, the epithelial island protects the thin apical cornea from UVA. Mazzotta *et al.* followed-up a small series of patients who underwent this technique and found it to be safe and efficacious (Mazzotta and Ramovecchi, 2014). Cagil *et al.* subsequently performed this technique on 19 eyes and observed an improvement in uncorrected visual acuity, best corrected visual acuity, flattest keratometry value and steepest keratometry values 1 year postoperatively. However, there was significant endothelial cell density loss (2550 ± 324 vs 2030 ± 200 cells/mm^2^) 1 year postoperatively (Cagil et al., 2017).

### Epi-Off-Lenticule-on Corneal Cross-linking

Sachdev *et al.* have reported three cases of successful CXL performed on thin, de-epithelialised corneas overlaid with a donor corneal lenticule. The refractive lenticules were obtained from patients who had undergone small incision lenticule extraction (SMILE). Instead of being applied directly onto the debrided corneal epithelium, this protocol requires riboflavin to be applied on a donor corneal lenticule. In this case series, this technique was found to be safe and effective (Sachdev et al., 2015). A study by Cagini *et al.* of this technique showed that visual acuity and endothelial cell density remained stable over a 12 month follow-up duration. Moreover, the presence of a demarcation line was seen in all patients by 6 months of follow-up (Cagini et al., 2020).

### Pachymetry-Based Accelerated Cross-Linking

Mazzotta *et al.* have proposed the M normogram, which puts together all available high-quality evidence on the depth of the demarcation line achieved across varying cross-linking protocols. This allows surgeons to choose from a list of protocols (of varying UVA regime and riboflavin formulation) to achieve the desired depth of cross-linking to minimise the risk of inducing endothelial cell toxicity. The M nomogram was validated against clinical findings of 20 eyes. Of these, eyes treated with 3 mW/cm^2^ conventional protocol showed an average demarcation depth of 350 ± 50 μm, while eyes treated with 30 mW/cm^2^ continuous light accelerated CXL had demarcation depths of 200 ± 50 µm. Additionally, eyes treated with 30 mW/cm^2^ pulsed light accelerated CXL had depths at 250 ± 50 µm while eyes treated with 15 mW/cm^2^ pulsed light accelerated CXL had depths at 280 ± 30 µm. Comparison of the measured demarcation line depths showed a high correlation between the measured and calculated depth based on the M nomogram (m = 1.03, *R* ^2^ = 0.73) (Mazzotta et al., 2018). However, this protocol has a distinct limitation - it requires surgeons to have access to various riboflavin formulations, and cross-linking devices that can output UVA energy at 3, 9, 15, and even 30 mW/cm^2^; using either continuous light or pulsed light protocols. Moreover, iontophoresis may also be required in some cases to perform the treatment.

### Sub400 Protocol

A study by Hafezi *et al.* introduced the sub400 individualised fluence CXL protocol which allows CXL to be performed on corneas with thickness of less than 400 µm (Hafezi et al., 2020). This aimed to circumvent disadvantages of reduced cross-linking efficacy conferred by earlier approaches that induced corneal swelling through hypoosmolar riboflavin or contact lens-assisted corneal cross-linking, and the need for specialised equipment for techniques such epi-Off-Lenticule-on and pachymetry based corneal cross-linking (Stojanovic et al., 2014).

Instead of modifying cornea thickness or riboflavin formulation, the sub400 protocol adjusts the UV illumination time and irradiance according to the corneal thickness to achieve a safe depth of cross-linking 70 µm away from the endothelium. The algorithm was published in 2017 and based on this algorithm, a pilot study of 39 eyes with corneal stromal thickness ranging from 214 μm to 398 μm showed that 90% of eyes established topographical stability at 12 months, while no eyes experienced endothelial decompensation (Kling and Hafezi, 2017). There was a significant improvement in corneal maximum keratometry values (−2.06 ± 3.66 diopters), but there were no changes in corrected distance visual acuity (Hafezi et al., 2020). This individualised approach is a promising new addition to existing treatment modalities for thin corneas, especially since this can be accomplished using standard CXL equipment.

## CXL and PACK-CXL at the Slit-Lamp (C-Eye Device)

Conventionally, PACK-CXL must be performed by corneal surgeons in a sterile operating theatre. C-Eye (EMAGine, Switzerland) is a miniaturised UVA irradiation system that can be mounted on a slit-lamp and operated by ophthalmologists in an ophthalmic clinic (Hafezi et al., 2021a). This device may provide better patient comfort because it allows patients to remain seated in front of the slit-lamp during the procedure. Epithelial debridement is performed by applying 40% ethanol over the central epithelium. Following which, riboflavin solution is applied, and UVA irradiation is administered. An *ex-vivo* study has demonstrated that this upright position that the patient adopts during UVA irradiation does not affect riboflavin distribution, with gravitational influence on riboflavin distribution only observed after 60 minutes of vertical positioning, well above the requirements of cross-linking which typically takes between 3 and 30 minutes (Salmon et al., 2017). The C-Eye device can also be used to perform cross-linking procedures for various corneal ectasias. This device may allow PACK-CXL to be better integrated as a treatment for infective keratitis during clinical care and allow PACK-CXL to be conducted even in resource-limited settings.

## Other Indications for Corneal Cross-Linking

### Combined Refractive Surgery With Corneal Cross-Linking (LASIK Xtra and SMILE Xtra)

Laser *in situ* keratomileusis (LASIK) and small incision lenticule extraction (SMILE) are procedures utilised to treat refractive errors. Simultaneous use of CXL with SMILE or LASIK in the same sitting is termed LASIK Xtra or SMILE Xtra and is postulated to strengthen the cornea postoperatively and reduce the development of postoperative corneal ectasia.

A study examining LASIK Xtra and SMILE Xtra protocols in LASIK rabbit ectasia models showed that mean curvature values decreased significantly following treatment. Furthermore, mean posterior elevation post-LASIK Xtra was found to be reduced compared to conventional LASIK (Konstantopoulos et al., 2019). While safe, two prospective randomised-controlled trials of LASIK Xtra vs. conventional LASIK have shown variable results. A study by Kanellopoulos *et al.* observed improved refractory and keratometric stability after LASIK Xtra (Kanellopoulos and Asimellis, 2015), while a study by Kohnen *et al.* showed no significant advantage in visual acuity, refraction or keratometric measurements with LASIK Xtra at 1 year follow-up (Kohnen et al., 2020). Liu *et al.* examined patients who underwent SMILE Xtra and found that its safety, efficacy, predictability, and stability are comparable with those of Femtosecond-LASIK Xtra at 1 year follow-up (Liu et al., 2021).

### Hyper-osmolar Riboflavin for Treatment of Bullous Keratopathy

CXL has been described in the management of patients with bullous keratopathy. Modifications to the riboflavin formulation includes use of intraoperative glycerol 70% or preoperative use of glucose 40% to dehydrate and reduce corneal oedema in patients with bullous keratopathy (Wollensak et al., 2009; Hafezi et al., 2010). In both studies, a reduction in central corneal thickness and best corrected visual acuity was observed, with no further episodes of bullae rupture and no re-intervention required postoperatively.

## Contraindications and Complications

### Contraindications to Corneal Cross-linking

Several contraindications for CXL have been suggested. These include pregnant or breastfeeding patients, patients with prior herpetic infections (due to the risk of herpes simplex virus reactivation), active ocular inflammation, severe central or paracentral corneal scars, autoimmune disorders, and a history of poor epithelial wound healing. Corneal thickness of less than 400 µm is increasingly considered a relative contraindication as it can be circumvented by utilising various techiniques described above.

### Transient Corneal Haze

Transient corneal haze after CXL is not uncommon. A study by Koller and colleagues prospectively evaluated 117 eyes which had undergone CXL and found that all eyes had anterior stromal haze at 1 month which subsequently improved within a 1 year period (Koller et al., 2009). Another randomised control trial of 36 patients with keratoconus found that the mean densitometry peaked at 1 month after treatment, but subsequently returned to baseline values after 6 months (Kim et al., 2016). Transient corneal haze is postulated to occur secondary to increased collagen fibre diameter post-CXL (Kozobolis et al., 2016). This is not typically associated with visual disturbances and often regresses after 12 months.

### Persistent Corneal Haze

On the other hand, persistent corneal haze lasting beyond 12 months should be differentiated from transient haze as this can negatively impact visual acuity and may respond to intensive topical steroid treatment (Mazzotta et al., 2015). This is thought to be secondary to the ongoing keratectasia process and corneal remodelling. A cohort study consisting of 34 eyes performed by Raiskup *et al.* observed 13 eyes (38.3%) with persistent corneal haze over a follow-up period of 10 years. (Raiskup et al., 2015). Preoperative risk factors identified include age over 35 years advanced keratoconus with minimal corneal thickness <400 µm and presence of preoperative activated keratocytes in the anterior stroma when measured with confocal microscopy. Intraoperative risk factors include forward defocus of UVA source, lack of riboflavin 0.1% application during irradiation and excessive riboflavin-dextran 20% solution causing stromal dehydration. Postoperative risk factors reported include non-compliance with postoperative topical corticosteroid therapy, infective keratitis, therapeutic lens intolerance, and presence of Langerhan cells after contact lens removal postoperatively (Mazzotta and Caragiuli, 2014; Mazzotta et al., 2015; Kim et al., 2016).

### Infectious Keratitis

Multiple incidences of post-CXL infectious keratitis have been reported. Offending organisms included Gram-negative organisms (*escherichia coli*, *pseudomonas aeruginosa*), Gram-positive organisms (*staphylococcus aureus*, streptococcus species), fungi and herpes simplex virus (Pollhammer and Cursiefen, 2009; Sharma et al., 2010; Al-Amry et al., 2017; Sitaula et al., 2019; Kodavoor et al., 2020). A recent study by Tzamalis and colleagues showed that postoperative application of bandage contact lens and topical steroids were independent risk factors for the development of microbial keratitis (Tzamalis et al., 2019).

The precise mechanism for herpes simplex virus reactivation after CXL remains unknown. It is postulated that emotional stress, trauma, and exposure to UVA are possible mechanisms of reactivation (Evangelista and Hatch, 2018).

### Other Complications

Further risks associated with CXL vary depending on the protocol used. In addition to corneal haze and post-CXL infectious keratitis, other complications include the development of a persistent epithelial defect, stromal scarring, corneal melt, corneal endothelial decompensation and damage to the lens or retina (Wollensak et al., 2003a). Development of late onset peripheral ulcerative keratitis following CXL has also been reported (Chanbour et al., 2019).

## Future Developments

A few areas are of particular interest. First, the dose-response relationship of UVA irradiation and riboflavin remains unclear. Although mathematical models have been postulated, their accuracy remains controversial as the exact molecular interactions of CXL remain elusive. Understanding the relationship between UVA, riboflavin and oxygen availability will enable clinicians to modify protocols in an informed and safe manner.

Second, in complementing the increasing utility of CXL as an anterior segment therapy, diagnostic tools are continually proposed to pick up subclinical ectasias. Corneal epithelial mapping is a procedure that is currently in vogue. It measures corneal epithelial and stromal thickness to identify areas of hypertrophy or thinning associated with corneal ectasias (Li et al., 2016). Machine learning algorithms which identify subclinical keratoconus have also been developed based on these measurements and are currently undergoing refinement (Shi et al., 2020). This will be useful in the identification and monitoring of progression, which may assist ophthalmologists in formulating recommendations for early corneal cross-linking.

Finally, indications for riboflavin as a photosensitiser for cross-linking have expanded beyond corneal ectasias. It is currently studied for use in correcting low myopia; and among post-radial keratotomy patients to reduce diurnal visual fluctuations (Elbaz et al., 2014; Elling et al., 2018).

## Conclusion

Advancements in CXL have changed the way we approach and manage keratectasias and a range of corneal diseases. The molecular mechanisms of CXL and its impact on corneal biomechanics continue to be extensively studied, with the hope that improvements can be made that enhance the efficacy and safety of this treatment. The advent of individualised UVA irradiation patterns may pave the way for safer and targeted treatment for keratoconus in the near future. Larger, long-term studies are required to validate the efficacy of new CXL techniques, but with the rapid accumulation of knowledge and experience in the field, CXL has become a robust treatment option for corneal ectasias, and is well primed to become a useful addition to our armamentarium for treating infectious keratitis and potentially in the future, even small refractive errors.
